# The Increasing Prevalence of Children Home Alone in Ghana: The Importance of Considering Regional Inequalities

**DOI:** 10.1007/s12187-023-10038-w

**Published:** 2023-05-27

**Authors:** René Iwo, Mónica Ruiz-Casares, José Ignacio Nazif-Muñoz

**Affiliations:** 1 University of North Carolina at Chapel Hill, Chapel Hill, NC USA; 2 McGill University, Montreal, Quebec Canada; 3 Toronto Metropolitan University, Toronto, Ontario Canada; 4 Université de Sherbrooke, Sherbrooke, Quebec Canada; 5 Centre de recherche Charles-Le Moyne, Longueuil, Quebec Canada

**Keywords:** child supervision, child wellbeing, parental supervision, home alone, Ghana

## Abstract

Research from industrialized settings has linked inadequate child supervision with various negative consequences. Nevertheless, empirical research in lower- and middle-income countries about correlates of inadequate child supervision has been scarce. The few studies that exist tended to focus on individual- and household-level factors, and reported associations that are not significant or in mixed directions depending on the context. Structural factors are left underexplored, but taking a more macro-level lens in settings with high regional disparities can hold the key to explaining increases in prevalence of inadequate child supervision. Exploring the evolution over time of child supervision practices can also enrich this explanation. We use data from two rounds of Ghana Multiple Indicator Cluster Surveys to examine factors associated with children left home alone, and employ regional analysis using strata-level mixed effects. We found that in Ghana, the prevalence of children left home alone without adult supervision increased by 8.5% between 2011 and 2018 – an increase of more than 500,000 children over seven years. Statistical analyses suggest that variation between regions likely are associated with the growth of inadequate child supervision in this country. Future research should pay closer attention to how structural conditions, proxied by regions, can serve as either barriers or facilitators to adequate child supervision practices, helping shed light on residual variance unexplained by individual- and household-level factors.

**Supplementary Information:**

The online version contains supplementary material available at 10.1007/s12187-023-10038-w.

## Introduction

Many young children around the world spend time at home alone or in the care of another young child, with no adults present (Ruiz-Casares et al., 2018). Inadequate supervision of children has been linked to various negative consequences, including higher risk of unintentional injuries (Halawa et al., 2015; Hyder et al., 2008; Morrongiello et al., 2013), sexual abuse (Ji et al., 2013), poorer academic performance, and behavioural and mental health problems (Akers et al., 2011; Dubowitz et al., 2005; Norman et al., 2012). While this arrangement is considered reportable child neglect in many industrialized countries (Coohey, 2003), it is a common practice in low- and middle-income countries (LMICs; Ruiz-Casares et al., 2018). There might be structural barriers to providing full-time adult supervision of children in these settings, such as lack of accessibility of childcare options (Miconi et al., 2018). Moreover, children supervising other children (with or without adults present) might even be seen as preparing older siblings into culturally normative roles (Levine et al., 1996) while freeing up adults to work for a living. Nevertheless, empirical research about this in LMICs has been scarce and population-based data on this phenomenon are limited (Miconi et al., 2018).

Among LMICs, countries in West and Central Africa show the highest prevalence of children under 5 years of age left home alone (ranging from 35.3% in Chad to 7.5% in São Tomé and Príncipe). Ghana figures in the middle of this, at around 16.5% of children under 5 years surveyed in 2011 (Ruiz-Casares et al., 2018). Ranking 138 out of 189 countries in Human Development Index (UNDP, 2019), Ghana has enjoyed steady economic growth over the last 15 years and successfully met their target of halving poverty from 1990 levels (Agyire-Tettey et al., 2021). Despite that, inequality remains large and the gap between rich and poor is growing both within and across regions and urban/rural areas (Agyire-Tettey et al., 2021; Cooke et al., 2016; Kangmennaang et al., 2019).

Many studies of correlates of child supervision focus on individual characteristics of children and their families. Overall, a study by Ruiz-Casares et al. (2018) of child supervision across 61 LMICs reported increased likelihood of children under 5 years being home alone among children who are older, have less educated mothers, and live with fewer adults in the household. However, depending on the country, patterns about the child’s sex, number of children in the house, wealth, and urban residence were mixed. Largely, similar patterns were observed for Ghana in 2011 in that study: non-adult child supervision is more likely among children who are older, have less educated mothers, and have fewer adults in the household. No significant associations were reported for child’s sex, number of children, wealth, and urban residence. While this study mapped a complex situation across countries at one single time point using individual and household characteristics, a more in-depth analysis at the country level is needed. One study done in Laos found large and important variation across provinces and urban/rural residence in the prevalence of children home alone (Ruiz-Casares & Nazif-Muñoz, 2018). Useful insights can be gained by exploring both whether structural or regional differences within countries can be associated with changes in the prevalence of this practice, and how this prevalence evolves over time. In this paper, we examine changes over time, 2011 and 2017/18, in the prevalence and factors of children under age 5 being home alone without adults present, and unpack these trends through a regional analysis.

## Child Supervision

### Background on Child Supervision in Ghana

Non-adult child supervision is legally considered as reportable child neglect in many countries (Coohey, 2003). While child neglect constitutes a social problem, one must develop a culturally relevant understanding of such issues, considering traditional and contextual dimensions (Ayim et al., 2023). Definitions and thresholds of what constitutes child neglect, along with perceptions thereof, vary widely from one population group to another. These are heavily influenced by culture, community beliefs, and norms (Abdullah et al., 2020; DeLong-Hamilton et al., 2016). Childcare practices seen as deviant in one culture might not be in another (Sawrikar, 2019). In fact, while child neglect (as defined according to the Western social work tradition) is common in Ghana, many Ghanaians might not see this as a major issue (Abdullah et al., 2020; Ayim et al., 2023). Non-adult child supervision might be a traditional cultural practice, socializing older children to normative caretaking roles or relying on community support in minding children who are home without adults present (Ayim et al., 2023; Castillo et al., 2015; Weisner, 1987). Moreover, childcare in many African countries is seen as a collective communal responsibility, both within and outside the nuclear family unit (Verhoef, 2005). Therefore, the issue of non-adult child supervision is culturally more complex and might offer some locally valued benefits.

In Ghanaian cultures specifically, childcare is traditionally perceived as the sole responsibility of mothers (not fathers) or kin and community (Abdullah et al., 2020; Ayim et al., 2023). Cultural beliefs of the fathers’ detached role in childcare might contribute to non-adult supervision of children, as the burden of supervision falls upon mothers alone, who might have other responsibilities. Furthermore, kin and community support might contribute to mothers assuming that their children are safe in the community’s hands (Abdullah et al., 2020), and do not require direct adult supervision at all times. This assumption is amplified in rural and northern areas of Ghana, where communal and kin-based living arrangements prevail (Abdullah et al., 2020; Nukunya, 2016).

### Individual and Household-level Correlates of Child Supervision

According to a scoping review on child supervision in LMICs by Miconi et al. (2018), most studies focused on the consequences and outcomes of differing child supervision practices. Relatively few investigated correlates (facilitators and barriers) of adequate supervision. The few studies in this latter category tended to center analyses on child, family, and household characteristics. At the child-level, their own capacity for independence, related to their age, is often cited as a factor in caregivers’ decisions around child supervision (Miconi et al., 2018). This capacity for independence is also related to whether the child is still breastfeeding. Breastfeeding children requires more time with mothers, thus making children less likely to be unsupervised.

Availability of and attendance at early childhood education (ECE) and care programs were also touted as factors that may have considerable effects on child supervision (Ruiz-Casares & Heymann, 2009). Such programs provide supervision for children where otherwise adults might have to leave children unsupervised. In fact, policies that allocate economic resources or social services such as these can have large impacts on supervision practices (Miconi et al., 2018). The Ghanaian government has greatly expanded ECE program facilities in public schools in the last decade or so (Agyire-Tettey et al., 2021), which might have an impact on child supervision practices.

Urban versus rural residence might also play a role in determining child supervision practices. The types of jobs available are different between the two areas: agriculture is more prevalent in rural areas, whereas manufacturing and formal jobs are more common in urban areas (Al-Hassan & Diao, 2007; Tanaka et al., 2018). The latter might not be as friendly to having children tag along their parents. Moreover, access to ECE and care programs might be more scarce in rural areas (Agyire-Tettey et al., 2021). Lastly, residential patterns, including density, proximity of kin, and sense of trust and safety within the neighbourhood likely factor into caregivers’ decisions regarding leaving a child at home without adult supervision (Ruiz-Casares & Heymann, 2009). These residential patterns differ between rural and urban areas. For instance, while density might be higher in urban areas, proximity of kin might be more likely in rural areas (Abdullah & Emery, 2022).

Family characteristics, especially surrounding socioeconomic conditions, are likely to also have an impact on supervision of children. Firstly, rapid economic changes (especially industrialization and urbanization) have been linked to drastic changes in family dynamics, such nuclearization of families as they migrate and labour force participation of women (who are traditionally the caregivers of children in many societies; Backhaus and Loichinger, 2022). These changes can hinder adequate adult supervision of children, especially if economic development in the region is not balanced by adequate policies that promote families’ and children’s wellbeing (Miconi et al., 2018). Moreover, material deprivation often leads to caregivers and adults in the household having to prioritize work outside the home that might not allow children to be around, leaving them unsupervised (Coope & Theobald, 2006; Ruiz-Casares & Heymann, 2009).

Previous research has found that parental education is related to child supervision practices in LMICs, in that more educated parents are less likely to leave their children home alone (Ruiz-Casares et al., 2018). Parents’ education is also related to their beliefs about what adequate parenting and supervision looks like (Robinson et al., 2018), and less educated parents might be less aware of the risks of inadequate supervision (Miconi et al., 2018). Furthermore, parental education might also reflect access to information and resources that helps parents access, prioritize, and afford childcare services in instances where they are not able to provide care themselves (Cuartas, 2022; Le & Nguyen, 2020).

Lastly, household composition has been found to have correlations with children being without adult supervision. Families with a higher number of older children in the home might have more leeway to leave children younger than 5 years old unsupervised by adults at home. A study in Botswana, Mexico, and Vietnam found that families where young children are occasionally or regularly left at home without adult supervision rely on older children to help with child care (Ruiz-Casares & Heymann, 2009). Furthermore, evidence on the relationship between the number of adults living in the home and child supervision practices is mixed. Living arrangements with extended families have been found to reduce caregiving responsibilities of primary caregivers and facilitate adult supervision of children (Miconi et al., 2018; Zevalkink et al., 2008). However, at the same time, non-nuclear living arrangements can either increase burden on the household’s finances, or reflect a strategy to weather economic hardship. In the latter case, more adults in the household might not necessarily be associated with better adult supervision of children, as the adults would likely be under more economic pressure.

### The Need for Regional Analysis

Most studies of correlates of child supervision tend to concentrate on individual characteristics of children and their families or households, and the quantitative evidence on this is often mixed depending on the context (Miconi et al., 2018). However, factors that prevail at the regional level are underexplored. Regions are potentially an important part of the explanation of disparities in child supervision practices, as they represent structural conditions: one region can be assumed to experience more similar exposures and “shocks” (for instance, in terms of socioeconomic changes or extreme weather events) than across regions. Each region in Ghana also comes with unique circumstances and characteristics, such as the northern regions having a higher concentration of subsistence agriculture with poorer soil quality compared to the southern regions (Al-Hassan & Diao, 2007). Thus, shocks such as droughts and flood disasters not only affect solely certain regions in the country, but they also affect each region differentially (Atanga & Tankpa, 2021). It is also important to note that cultural practices, including about childcare, differ across the more than 70 ethnic groups in Ghana (Ayim et al., 2023), and each ethnic group tends to cluster within specific regions (Agyei-Mensah & Owoo, 2015). These regional differences in ethnic composition also play a role in creating variations between regions in terms of childcare practices. Thus, conducting regional analyses may help to shed light on the residual variance in child supervision practices that remain unaccounted for by individual child and household-level factors.

Economic development in Ghana in the past decade and a half has been rapid and effective at reducing poverty (Agyire-Tettey et al., 2021), but regional inequalities remain (Cooke et al., 2016). With diverse terrain and peoples, Ghana was subdivided into 10 regions until a referendum in 2018 split up some of the larger regions and created 6 new ones (Shaban, 2018). There is a stark north-south divide in development in which the north lags behind the south: poverty rate in 2013 remained around 50% in the three northernmost pre-referendum regions (Northern, Upper East, and Upper West), while the national rate was at 24.2% (Cooke et al., 2016; Tanaka et al., 2018). Poverty reduction has stagnated in these regions, and the absolute number of poor people has increased in the last few years (Tanaka et al., 2018). The government has implemented various social and economic development policies and programs (Al-Hassan & Diao, 2007; Ghana Statistical Service (GSS), 2016; UNICEF, n.d.). Nevertheless, their implementation requires further attention, especially as it might actually have the effect of worsening disparities between regions where the programs are effectively implemented and other regions where implementation is much poorer (Tanaka et al., 2018). For example, nationwide farmer support programs helped alleviate poverty in the southern regions but not as much in the northern regions, as the implementation does not take into account the different soil and topography of the north (Al-Hassan & Diao, 2007). And while the driver of poverty alleviation nationally has been expansion of trade, northern Ghana has not benefitted from this as much as the south (Al-Hassan & Diao, 2007).

These regional inequalities in Ghana produce varied structural factors that are likely to mirror different child supervision practices. However, not much research has explored associations at this more macro level. Our study aims to contribute to the child supervision literature by looking at its correlates at multiple levels: we start by replicating previous studies using individual factors at the child and family or household level, then we conduct a regional analysis.

## Methods

### Data and Variables

We used data about children aged below 5 years in Ghana’s Multiple Indicator Cluster Survey rounds 4 (MICS 4, 2011, N = 7,550 children) and 6 (MICS 6, 2017/18, N = 8,879 children). These were the two latest rounds of the survey data available (MICS 5 does not exist for Ghana) that included our outcome variable of interest. MICS is a repeated cross-sectional household survey that includes information about health, development, and child protection through face-to-face interviews. As sampling strategies of both survey rounds are similar, analyses across rounds of surveys are thus feasible, allowing the production of trend data of changes at the societal level (Khan & Hancioglu, 2019). These surveys use the most recent Ghana Population Censuses as their sampling frame, and are nationally representative of children aged 0–4. The surveys have remarkably high response rates of 99% and 99.7% for MICS 4 and 6 respectively (Ghana Statistical Service (GSS), 2011, 2018). Several regions were oversampled (Central, Northern, Upper East, and Upper West)^1^, but the application of sample weights corrects for the sampling design and oversampling (Ghana Statistical Service (GSS), 2011, 2018)

Our outcome measure of interest was *the number of days a child was home alone for more than an hour in the past week*, derived from a question that asks primary caregivers, “Sometimes adults taking care of children have to leave the house to go shopping, wash clothes, or for other reasons and have to leave young children. On how many days in the past week was [child’s name] left alone for more than an hour?” Answers ranged from 0 if the child was not left home alone, and 1 to 7 depending on the number of days the child was left home alone. We used this information from MICS 4 and 6 to examine changes of child home alone over time, and the factors associated with any such changes.

Measures of child and household characteristics were used as independent variables. Child characteristics variables included *sex* and *age* (in months) of the child; as well as dichotomous markers of whether the *child currently breastfeeds* (ages 0–2 only) and whether the *child attends any ECE programs* (ages 3–4 only). At the family level, we included *urban/rural residence*; and *wealth quintiles* derived by MICS from factors such as house characteristics (i.e., electricity, water facilities, number of rooms, type of toilet, and building material) and presence of material goods (i.e., television, telephone, refrigerator, computer, camera). The lowest quintile represented the poorest group and the highest the richest. We also included *mother’s* and *father’s years of education* as another marker of socioeconomic status and differential approaches to childrearing. The variables mother’s and father’s education were only moderately correlated (Pearson’s r = 0.53), thus inclusion of both in the same model does not pose multicollinearity problems. As for household composition, we included the *total number of children aged 5–17 years* and *total number of adults aged 18 years and older* living in the household. We also added a dichotomous variable marking the *year or round of survey* (2017/18 for MICS 6, ref. 2011 for MICS 4).

### Analysis

We applied Poisson regression models to estimate the correlates of the number of days children were left home alone. This is the most appropriate modelling strategy for our outcome of interest, which is a count variable of number of days (0–7). We used robust variance to account for the multistage stratified cluster sampling design with sampling within the 10 regions of Ghana. These correspond to the extant subdivisions at the time of the survey, before the 2018 referendum on creating new regions (Shaban, 2018). We applied the appropriate weights to provide estimates that are generalizable to the population of children aged under 5 years in Ghana at the time of survey. These weights were constructed based on the most recent Ghana Population Censuses. They were provided in the data to account for probability of selection at every stage of sampling, as well as non-response at the household and individual levels. More information is available in the MICS reports for each survey (Ghana Statistical Service (GSS), 2011, 2018). We also conducted model diagnostics to ensure that there are no problems related to multicollinearity (variance inflation factors) and model specifications (linktest). We found that our models show none of these issues.

We pooled both survey rounds but stratified regressions by age group, namely ages 0–2 and 3–4 years. For each age group, we built the models sequentially by adding more variables at each step. All models included a variable marking the survey year/round. We started with child characteristics: sex and age of child, and breastfeeding (for ages 0–2 years) or current attendance at any ECE programs (for ages 3–4 years). Next, we added the family characteristics variables, namely urban/rural residence, wealth index quintiles, and mother’s and father’s years of education. Then we included variables for household composition into the model. All analyses are done using STATA 16 software. Coefficients are exponentiated by the software and reported as incidence rate ratios (IRR), with 95% confidence intervals (CI). This was done for ease of interpretation. For example, an IRR of 2 indicates an incidence rate twice as high compared to the reference group, which is more readily comparable than IRR’s unexponentiated counterpart (log counts).

Specifically for ages 3–4 years, we added an interaction term between survey round and child attendance at any ECE program, to test whether the continuously expanding accessibility of ECE programs in Ghana played a larger role in predicting the outcome variable in 2017/18 (MICS 6) compared to 2011 (MICS 4).

Lastly, considering previous concerns about regional and urban/rural inequalities in Ghana, we re-estimated the fully adjusted model as a multilevel mixed-effects Poisson regression accounting for clustering within strata of the survey (20 strata: 10 regions of Ghana, each divided into urban and rural). A large change in the strata mixed-effects model would signal whether much of the variance was at the strata level, indicating differences between regions and urban/rural areas. This serves as a basis to examine differences between regions more specifically, by adding an interaction between the dataset source and the 10 regions to examine more localized changes over both survey rounds. We present predicted probabilities of the outcome variable in a graph, disaggregated into the 10 regions of Ghana for each survey round.

## Results

Table 1 presents weighted descriptive statistics of variables used. Prevalence of children home alone in Ghana was 16% in 2011 (MICS 4) but was higher six years later at 24% in 2017/18 (MICS 6). This difference was statistically significant (χ^2^-test p < 0.000). While breastfeeding stayed largely stable at around 57% when comparing both survey rounds, the percentage of children attending any ECE programs rose slightly from 67% to 2011 to 71% in 2017/18 (χ^2^-test p < 0.000). The percentage urban and wealth distribution seemed largely stable over time. Both mother’s and father’s education improved slightly between the two time points, with a more modest increase for fathers than mothers. In both survey rounds, fathers had slightly higher number of years of completed education than mothers. The number of older children (ages 5–17) and adults (ages 18 and above) in the house rose between the survey rounds.

**Table 1:** Weighted descriptive statistics of variables associated with children aged below 5 years left home alone in Ghana, MICS 4 (2011) and MICS 6 (2017/18)

	MICS 4 (2011)			MICS 6 (2017/18)	
Variable	Mean or %	SD (range)		Mean or %	SD (range)
Number of days home alone in past 7 days	0.529	1.443 (0–7)		0.769	1.673 (0–7)
0 in the past 7 days	84.08%			75.60%	
1–7 days in the past 7 days	15.92%			24.41%	
3–7 days in the past 7 days	9.23%			12.03%	
5–7 days in the past 7 days	4.75%			6.51%	
Male (ref. *female)*	49.76%			49.21%	
Age of child (months)	29.13	17.225 (0–59)		30.04	17.190 (0–59)
Child breastfeeds (ages 0–2 only)	58.66%			56.73%	
Child attends education program (ages 3–4 only)	67.39%			70.79%	
Urban residence (ref *rural*)	43.5%			43.1%	
Wealth index quintile of household					
Quintile 1 (poorest)	22.92%			22.14%	
Quintile 2	20.54%			20.65%	
Quintile 3	20.64%			19.95%	
Quintile 4	18.51%			18.90%	
Quintile 5 (richest)	17.39%			18.36%	
Mother’s education (years)	5.25	4.563 (0–18)		6.09	4.603 (0–18)
Father’s education (years)	6.75	5.139 (0–18)		7.18	4.937 (0–20)
Missing	28.67%			38.71%	
Number of household members ages 5–17	1.89	1.712 (0–15)		2.26	1.897 (0–15)
Number of household members ages 18+	2.44	1.200 (1–14)		2.79	1.566 (1–17)
**N**	**7,527**			**8,786**	

Table 2 presents the regional breakdown of the sample, as well as the outcome variable of children home alone. The sample was quite evenly distributed between the different regions, except for the Northern region, where 19.3% of the sample resided. The Northern region was the largest region in Ghana by area, and hence had the largest number of clusters and enumeration areas. It was also oversampled, as mentioned above. As for mean number of days children were home alone in the past week, the highest in MICS 4 (2011) was observed in Brong Ahafo at 0.81 days, but this rate has gone down to 0.69 in MICS 6 (2017/18). The highest in MICS 6 was the Northern region at 1.71 days. The Western and Volta regions had figures that stayed unchanged between survey rounds. The other seven regions saw an increase in the mean number of days children were home alone, with three regions having increased at least twofold: the Eastern region increased from 0.26 to 0.58, the Northern region increased from 0.73 to 1.71, and the Upper East region increased from 0.49 to 1.03. Nationwide, the change was from 0.61 to 2011 to 0.83 days in 2017/18, an increase of about 32%.

**Table 2:** Regional breakdown of the sample and mean number of days child left alone in the past 7 days

		Number of days child left home alone in past 7 days			
Region	N (%)	Mean (SD) MICS 4 (2011)	Mean (SD) MICS 6 (2017/18)	Difference	Percentage change
Western	1,266 (7.9%)	0.63 (1.55)	0.62 (1.39)	-0.01	98%
Central	1,850 (11.3%)	0.53 (1.37)	0.60 (1.43)	0.07	113%
Greater Accra	1,156 (7.1%)	0.40 (1.20)	0.60 (1.53)	0.20	150%
Volta	1,175 (7.2%)	0.41 (1.28)	0.42 (1.17)	0.01	102%
Eastern	1,145 (7.0%)	0.26 (1.07)	0.58 (1.35)	0.32	223%
Ashanti	1,593 (9.7%)	0.55 (1.35)	0.68 (1.48)	0.13	124%
Brong Ahafo	1,229 (7.6%)	0.81 (1.95)	0.69 (1.62)	-0.12	85%
Northern	3,141 (19.3%)	0.73 (1.77)	1.71 (2.41)	0.98	234%
Upper East	1,724 (10.6%)	0.49 (1.34)	1.03 (2.08)	0.54	210%
Upper West	2,034 (12.4%)	0.75 (1.83)	0.99 (1.82)	0.24	132%
Ghana (nationwide)	16,313 (100.0%)	0.61 (1.58)	0.83 (1.74)	0.22	136%

Regression results for children aged 0–2 years are presented in Table 3. Model 1 included only child-level characteristics and showed that age of the child (in months) had a significant positive correlation with the number of days they were left home alone that remained robust across the different models. Sex and breastfeeding did not show significant correlations with the outcome. When family-level characteristics were included (model 2), wealth did not have any significant correlations with the children home alone, and only father’s education seemed to have a modest negative correlation with the outcome. This latter correlation persisted even after the household composition variables were added to the model (model 3), while composition of the household itself did not show any significant correlations. After including the strata mixed-effects, only the age of the child and the survey rounds variables had significant correlations with the outcome variable. In fact, this positive correlation between survey rounds and child home alone was consistent across the board, and remained significant at an IRR of 1.53 (95% CI = 1.10–2.13; model 4). Model 4 shows that a significant portion of the variance can be explained by clustering at the strata level, as evidenced by the significant strata-level variance. Based on the Bayesian Information Criterion (BIC), it appears that the mixed-effects model was the best fit (smallest BIC), pointing to the relevance of differences across strata.

**Table 3:** Weighted Poisson regression results estimating incidence rate ratios (IRR) of number of days children aged 0–2 were left home alone in the past 7 days, Ghana MICS 4 (2011) and MICS 6 (2017/2018)

	Model 1	Model 2	Model 3	Model 4
Male child (ref. female)	1.03	1.03	1.03	1.02
	(0.88, 1.20)	(0.88, 1.20)	(0.88, 1.20)	(0.85, 1.21)
Age of child (months)	1.05***	1.04***	1.04***	1.04***
	(1.04, 1.06)	(1.03, 1.06)	(1.03, 1.06)	(1.03, 1.06)
Child breastfeeds	1.03	0.90	0.90	0.88
	(0.80, 1.33)	(0.70, 1.17)	(0.69, 1.17)	(0.69, 1.12)
Urban residence (ref. rural)		0.88	0.89	0.99
		(0.73, 1.07)	(0.74, 1.08)	(0.74, 1.32)
Wealth index quintile of household (ref. Quintile 1, poorest)				
Quintile 2		0.85	0.85	0.87
		(0.68, 1.06)	(0.68, 1.06)	(0.70, 1.08)
Quintile 3		0.93	0.93	0.97
		(0.72, 1.20)	(0.73, 1.20)	(0.72, 1.32)
Quintile 4		0.94	0.94	0.98
		(0.69, 1.28)	(0.69, 1.28)	(0.69, 1.38)
Quintile 5, richest		0.98	0.97	0.98
		(0.71, 1.35)	(0.71, 1.33)	(0.71, 1.35)
Mother’s education (years)		0.98	0.98	0.99
		(0.96, 1.00)	(0.96, 1.01)	(0.97, 1.01)
Father’s education (years)		0.97*	0.97*	0.98
		(0.95, 0.99)	(0.95, 1.00)	(0.95, 1.00)
Number of household members ages 5–17			1.02	1.02
			(0.98, 1.07)	(0.98, 1.07)
Number of household members ages 18+			1.04	1.03
			(0.98, 1.09)	(0.99, 1.07)
2017/18 (ref. 2011)	1.55***	1.58***	1.54***	1.53*
	(1.31, 1.84)	(1.33, 1.88)	(1.30, 1.83)	(1.10, 2.13)
Bayesian Information Criterion	22253.12	22111.45	22100.11	21913.04
*Strata-level variance (standard deviation)*				0.07** (0.02)
**N**	**9,616**			

**Notes** Results presented in incidence rate ratio (IRR) form, 95% confidence interval in parentheses; * p < 0.05, ** p < 0.01, *** p < 0.001

Regressions results for children aged 3–4 years are presented in Table 4. We again see a consistently positive correlation between age of the child and the outcome of child home alone, but no significant associations were reported for sex. In model 1, attendance at an ECE program had a significant negative correlation with the outcome, but this was no longer statistically significant once family characteristics variables were added in model 2. Family characteristics were shown to have negative correlations with the outcome. Compared to the poorest, wealthier quintiles have progressively lower incidence of being home alone, with the wealthiest quintile having an IRR of 0.61 (95% CI = 0.46–0.81). Both mother’s and father’s education had a consistent significant negative correlation with children being home alone at the same IRR of 0.97 (95% CI = 0.95–0.99). In model 3, we observed that while the number of children aged 5–17 years in the household had no significant association with the outcome variable, the number of adults had a significant positive association (IRR = 1.05 for each increase of one adult, 95% CI = 1.00-1.10).

**Table 4:** Weighted Poisson regression results estimating incidence rate ratios (IRR) of number of days children aged 3–4 were left home alone in the past 7 days, Ghana MICS 4 (2011) and MICS 6 (2017/2018)

	Model 1	Model 2	Model 3	Model 4	Model 5
Male child (ref. female)	1.13	1.13	1.13	1.14*	1.12
	(0.99, 1.28)	(1.00, 1.28)	(1.00, 1.29)	(1.01, 1.29)	(0.98, 1.30)
Age of child (months)	1.02**	1.01*	1.01*	1.01*	1.01
	(1.01, 1.03)	(1.00, 1.02)	(1.00, 1.02)	(1.00, 1.02)	(1.00, 1.02)
Child attends education program	0.71***	0.98	0.98	1.18	1.17
	(0.62, 0.81)	(0.85, 1.12)	(0.85, 1.13)	(0.95, 1.48)	(0.90, 1.53)
Urban residence (ref. rural)		0.92	0.94	0.94	0.78
		(0.78, 1.09)	(0.79, 1.11)	(0.79, 1.11)	(0.58, 1.05)
Wealth index quintile of household (ref. Quintile 1, poorest)					
Quintile 2		0.81*	0.81*	0.81*	0.91
		(0.69, 0.96)	(0.68, 0.95)	(0.68, 0.95)	(0.78, 1.07)
Quintile 3		0.84	0.83	0.83	1.01
		(0.69, 1.03)	(0.68, 1.02)	(0.68, 1.02)	(0.79, 1.28)
Quintile 4		0.70**	0.70**	0.69**	0.86
		(0.55, 0.91)	(0.54, 0.90)	(0.54, 0.89)	(0.66, 1.12)
Quintile 5, richest		0.61***	0.60***	0.60***	0.75
		(0.46, 0.81)	(0.45, 0.80)	(0.45, 0.80)	(0.54, 1.05)
Mother’s education (years)		0.97**	0.98*	0.98*	0.99
		(0.96, 0.99)	(0.96, 1.00)	(0.96, 1.00)	(0.97, 1.02)
Father’s education (years)		0.97**	0.97**	0.97**	0.98
		(0.95, 0.99)	(0.95, 0.99)	(0.95, 0.99)	(0.96, 1.01)
Number of household members ages 5–17			1.01	1.01	1.00
			(0.97, 1.05)	(0.97, 1.05)	(0.95, 1.06)
Number of household members ages 18+			1.05*	1.05*	1.02
			(1.00, 1.10)	(1.00, 1.10)	(0.97, 1.07)
2017/18 (ref. 2011)	1.35***	1.42***	1.40***	1.69***	1.64**
	(1.18, 1.55)	(1.24, 1.62)	(1.23, 1.60)	(1.38, 2.07)	(1.20, 2.25)
2017/18 * attends education program				0.74*	0.74
				(0.57, 0.96)	(0.54, 1.01)
Bayesian Information Criterion	22998.18	22366.13	22352.57	22331.88	21616.6
*Strata-level variance (standard deviation)*					0.26* (0.12)
**N**	**6,691**				

**Notes** Results presented in incidence rate ratio (IRR) form, 95% confidence interval in parentheses; * p < 0.05, ** p < 0.01, *** p < 0.001

In model 4, we included an interaction term between survey round and the variable indicating attendance at any ECE program. While the IRR for survey round was then pushed up to 1.69 (95% CI = 1.38–2.07), this interaction term showed a rather large and significant negative correlation with the outcome variable (IRR = 0.74, 95% CI = 0.57–0.96). This shows that only in 2017/18 (MICS 6) did attending an ECE program become correlated with a significantly lower incidence of being left home alone, and not in 2011 (MICS 4). However, once the strata were considered in the mixed-effects model (model 5), none of these associations remained statistically significant, except for the positive correlation with the variable marking the survey round. Again, the mixed-effects model was shown to be the best fitting model by virtue of it having by far the smallest BIC, and the strata-level variance was shown to be significant. This again hints at the relevance of variation between strata in unobserved characteristics.

**Fig. 1:**
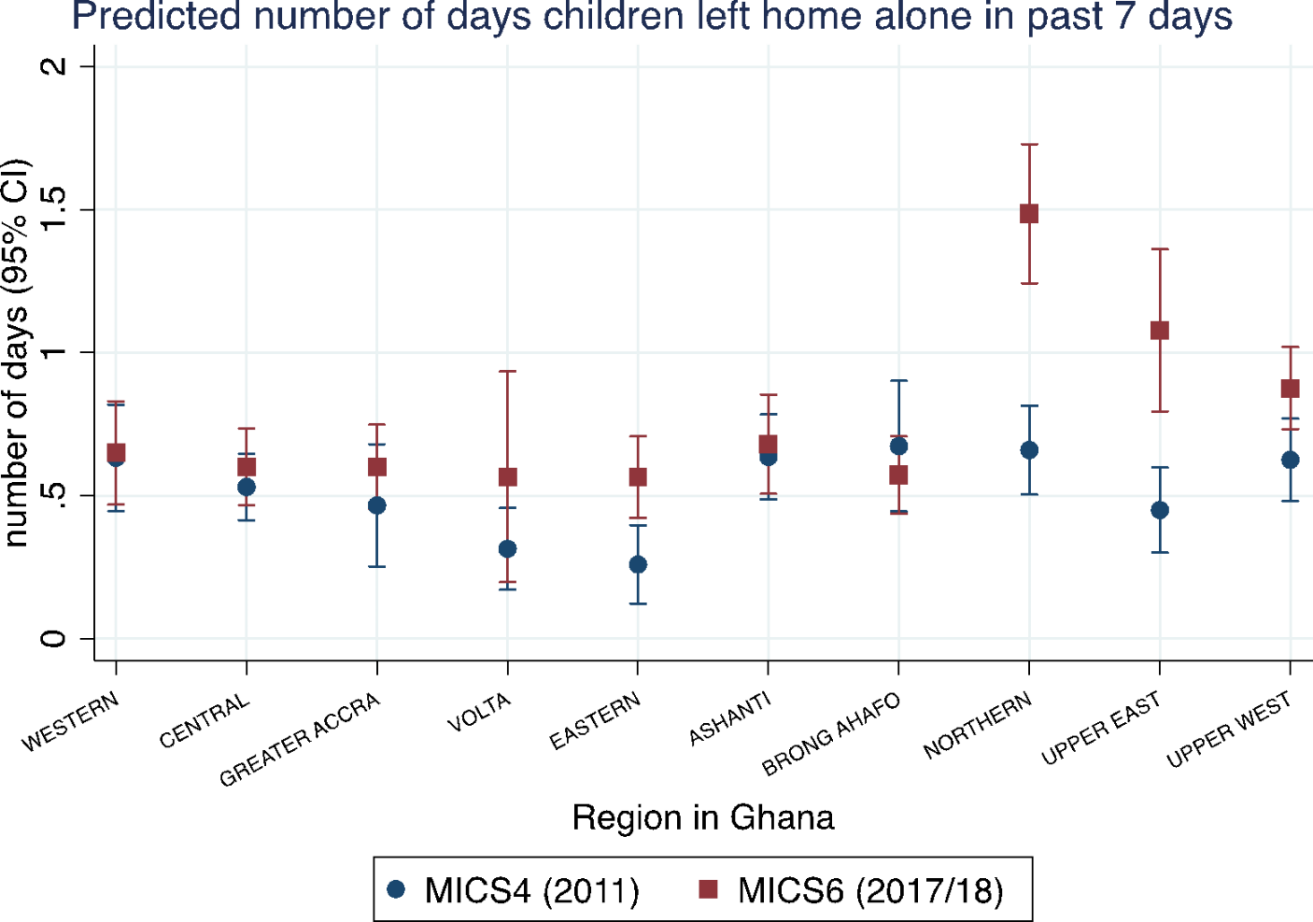
Predicted number (with 95% confidence intervals) of days children aged below 5 years left home alone in the past 7 days in Ghana (MICS 4 and 6)

Figure 1 shows predicted number of days of children home alone by all 10 regions and data sources (MICS 4 and 6). The figure illustrates how wide the variation was between regions, from 0.26 in the Eastern region in MICS 4 to 1.48 in the Northern region in MICS 6. The number was also higher in MICS 6 than 4 in almost all regions. MICS 6 showed significantly higher predicted number of days compared to MICS 4 in three regions (Eastern, Northern, and Upper East), similar to descriptions from Table 2. In the Eastern region, the predicted number of days a child was home alone was 0.26 in 2011 (the region with the lowest prevalence in MICS 4) to 0.56 in 2017/18, equivalent to 2.2 times higher in MICS 6. In 2017/18 in Northern and Upper East, the numbers were already substantially higher than other regions, at above one day per week. In the Northern region, it increased from 0.66 to 2011 to 1.49 in 2017/18 (2.3 times higher), while in the Upper East region it increased from 0.45 to 1.08 in between the same time points (2.4 times higher). These two regions had not been outliers in 2011 as they were in 2017/18. The Upper West region also showed numbers that were consistently higher than average, although the increase between 2011 and 2017/18 in this case was only marginally significant.

## Discussion

Between 2011 and 2018, prevalence of children left home alone in Ghana increased by 8.5% to almost a quarter of the population of children aged 0–4 years. Substantively, this means an increase of more than 500 thousand children (in absolute number of children, accounting for change in population sizes), amounting to 1.04 million children in Ghana having been left home alone at least once in the past 7 days in 2018^2^ (GSS, 2020). Although this calculation is provisional (see footnote 2), this increase was significant both statistically and substantively. Indeed, our regression results confirmed this, with the IRR for MICS 6 across the board being significantly positive and strong in its correlation with the number of days children were under non-adult supervision. This correlation remained strong and positive even after strata mixed-effects were included, suggesting that this correlation persisted within regions.

We found largely similar associations when comparing results of mixed-effects regressions (with strata as the second level) that are stratified by MICS 4 and MICS 6 (see Table S1 in supplementary materials) and found no conclusive differences in associations between rounds when including interaction terms for the survey round. Hence, we decided to pool the two survey rounds. We started our analysis by including individual-level characteristics of the child, then sequentially adding socioeconomic status variables and household composition variables into the models. Our analysis suggested that while there were a few significant associations at the individual or household level with child home alone, these associations were weak and disappeared once we added a strata-level mixed effect. The strata-level mixed effect models were shown to be the best fitting model according to the BICs. This points to the importance of regional-level differences in child supervision practices, hinting at a more structural explanation of disparity between regions.

As for the individual-level characteristics, we reported no distinct patterns for sex of child and being home alone, which might be because sex roles around the house and demands on their parents are barely differentiated for male or female children at ages 0–4 years. Moreover, we observed a strong pattern of older children being more likely to be home alone. This age pattern was also found in previous research including many LMICs. This might be attributed to late toddlerhood signifying the end of breastfeeding, as well as increasing weight and mobility which makes carrying or bringing children to work more challenging, thus leaving them at home (Ruiz-Casares et al., 2018; Ruiz-Casares & Nazif-Muñoz, 2018). For the younger age group, breastfeeding was associated with a modest incidence of being left home alone. This was expected as the mother must be with the child more often if they are still breastfeeding.

For older children, attendance at an ECE program showed the expected negative correlation with being home alone. While participating in an ECE program, the children would be under the supervision of the teachers, while their usual adult caregivers would be able to accomplish other tasks. This association lost statistical significance once family-level socioeconomic variables were considered. Given the presumed increasing availability and accessibility of ECE programs over time, we tested whether there was significant difference in 2017/18 in the correlation of ECE attendance with the outcome variable, above and beyond the IRR for 2011. We did this by adding an interaction term between the survey year and ECE program attendance. Only in 2017/18 did we see that ECE program attendance was associated with a significantly lower incidence of child left alone, while this association was not statistically significant in 2011. Perhaps the available ECE programs were not as accessible to those in the lower socioeconomic groups back in 2011, or the uptake of the extant offerings had been insufficient in making a difference in child supervision practices. Although the increase in ECE enrollment in our data was quite modest (from 67 to 71% between 2011 and 2017/18), the Ghanaian government’s success in expanding ECE programs over the last decade (Agyire-Tettey et al., 2021) may have had a lagged effect that was only emerging in 2017/18. However, we see that this correlation for the interaction term in 2017/18 is rendered not significant in the multilevel mixed-effects model. This might suggest that access to such programs (and the desirable impacts it might have on parental supervision) are not homogeneously distributed across regions.

In terms of family socioeconomic differences, previous research has shown that higher family resources and parental education is associated with certain parenting practices that are more intensive and involved, as well as valuing early education for their children (Robinson et al., 2018). As expected, our results demonstrated that more years of parental education and being in a higher wealth quintile generally showed lower incidence of child left alone. However, these correlations were only statistically significant for children aged 3–4 years. For children aged 0–2 years, only father’s education had a statistically significant negative correlation with the outcome, suggesting that father’s and mother’s education might correlate with the outcome variable through different pathways. Perhaps for younger children, it is more a question of resources, which might be better reflected in the father’s education level. Mothers, given cultural expectations of them being more highly involved in caring for an infant/toddler compared to older children, might parent more similarly regardless of education level. Whereas for older children, parenting practices might differ more substantially, which might explain why both mother’s and father’s education matters, as it might reflect differing approaches to parenting. Future research should explore more deeply the dynamics of human capital of fathers and mothers and how they might differ in terms of parenting and supervision practices.

Furthermore, we observed mostly no significant correlations between household composition and the outcome variable. The only exception was for children aged 3–4 years: the presence of more adults in the household was associated with higher incidence of children home alone. This questions previous research, where more adults in the home usually means lower likelihood of children home alone (Ruiz-Casares et al., 2018). We speculate that this seemingly counterintuitive pattern reflects either variation over time, or a growing prevalence of extended-family household units as an economic adaptation strategy, where more adults in the household work outside the home and contribute to the household’s finances (GSS, 2016). This growth in extended-family housing arrangements was parallel with our finding that household size (number of children aged 5–17 years and adults aged 18 years and above) had increased over time, as seen in Table 1.

Our mixed-effects models consider clustering within the 20 strata of the survey (10 regions of Ghana, each separated into urban and rural), and were shown to have the best fit. In these models, except for age, almost all correlations of individual and household-level factors appeared as non-significant. This points to most of the associations having been absorbed by variations between strata, suggesting that there was strong clustering within strata. The individual and family-level factors included were likely to be distributed unequally across different regions, as has been previously discussed (Agyire-Tettey et al., 2021).

We also included a regional-level variable, namely poverty headcount rate data from 2013 (mapped onto observations from 2011; Cooke et al., 2016) and 2016 (mapped onto observations from 2017/18; The World Bank 2018). We added this regional poverty rate variable at the second level (strata) of our multilevel mixed-effects Poisson models. (see Table S2 in supplementary materials). Results do not alter our conclusions about the importance of unobserved structural variables at the regional level in explaining the disparities in child home alone between regions. These unobserved structural variables may be related to the unique circumstances and characteristics of each region, such as composition of livelihoods, and/or exposure to shocks of each region. Ethnic groups also tend to cluster within regions (Agyei-Mensah & Owoo, 2015), and differing cultural practices between these groups are likely to play a role in creating regional variations in childcare.

In turn, it is thus important to unpack these trends by region and time. Indeed, we found three out of ten regions had significant increases in prevalence of child home alone: two of these were in the far north (Northern and Upper East) and were also the poorest regions of the country (Agyire-Tettey et al., 2021; Cooke et al., 2016). Meanwhile, the Eastern region saw larger growth in the proportion of poor population compared to other regions in recent years (Cooke et al., 2016). This potentially connects regional economic deprivation to a higher likelihood of children being left home alone, revealing a pattern at the ecological level that is independent to the individual and household-level variables and requires further investigation.

Overall, our regression models demonstrate how concentrating on regions diminished the explanatory power of individual and household-level factors substantially. However, such regional analyses with a macro-level lens are quite rare in the literature, as most studies focus on more micro-level factors to explain parenting and supervision practices. Our analysis underlines how future research should pay closer attention to how structural conditions, proxied by regions, can serve as either barriers or facilitators to adequate child supervision practices, on top of the individual and household-level factors.

Some research limitations must be noted. First, the survey design presents some limits to interpretation. As a repeat cross-sectional survey, the relationship between the exposure and outcome should not be interpreted as causal (Carlson and Morrison, 2009), and changes over time should not be interpreted as changes in individual behaviour. Second, as a household-based survey, children not residing in the household (e.g., in the streets or in residential care) were not captured. Third, the questionnaire was administered in English (in MICS 4) or four local languages (MICS 6) by trained enumerators. Yet, different interpretations of being “alone,” social desirability, or problems with recall cannot be ruled out. Fourth, parental availability for supervision was assumed constant within country and wave. However, data collection extended over three months and adult availability to supervise children may have fluctuated in that timespan (e.g., during harvesting periods, during holidays). Fifth, measurement error cannot be ruled out when comparing estimates by residence location as the realities of home life might not be properly captured by the survey item (e.g., poorer or urban households may actually be clustered together so that young children may not be out of sight of an adult, or rural areas might be perceived safer); qualitative studies into this issue might bring important insights.

Another caveat to this study is the fact that there are households with more than one child in the dataset. The observations of the outcome variable (number of days children home alone) is not always the same for all children living in the same household. This suggests that parents can have different approaches to caring for different children (who presumably are mostly of different ages), reflecting varying restrictions that they may face. Further qualitative research might be able to delve deeper into this intra-household variation. For our purposes, we conducted sensitivity analyses in two ways: clustering standard errors at the household level, and adding household as a second level in the multilevel mixed-effect Poisson models. Both showed that our results remained robust, as they did not change in terms of direction, approximate size, and significance of the associations.

Finally, MICS data did not collect information on parental employment, union separation, or whether/when new members join the household. As such, we were not able to explore the dynamics of these three factors on the outcome variable. Future studies should examine employment status of adult household members, changes in the household composition over time, and childcare arrangements in Ghana’s rapidly industrializing economy, as adults might have to change their child supervision practices as they obtain new kinds of jobs outside the home, migrate either with or without children (Ackah and Medvedev, 2010), experience divorce or a change in household composition and size. The relationship of unemployment with rurality and education should also be explored further as unemployment is generally higher for people with education than no education (although tertiary-educated adults are more likely to be employed) at the national level, yet the opposite is true in urban areas (Backhaus and Loichinger, 2022; GSS, 2016).

Despite these limitations, this study showcased the value of regional analyses to understand changes in the prevalence of children under five years home alone in Ghana between 2011 and 2018. The Ghanaian government has implemented multiple programs that might help adults stay home to care for children (e.g., family-based cash transfers and community-based health services), or provide supervision for children through institutions (e.g., two years of free and compulsory kindergarten; GSS et al., 2015; UNICEF, n.d.). While its implementation might not be equally effective across the whole country, perhaps there are confounding factors that impede the effectiveness of these programs on improving early childhood care and supervision. Continuous improvements in policies and program delivery by both the Ghanaian and regional governments would likely shift these structural factors, and hopefully decrease regional disparities in poverty and inadequate child supervision. Further studies incorporating economic, migration, and other structural factors are necessary to unpack this, perhaps drawing in regional or macro-level data from other sources. As our findings show, regional or structural factors may hold the key to explaining differences in parental supervision practices that cannot be explained by individual or household-level factors.

## Electronic supplementary material

Below is the link to the electronic supplementary material.

Supplementary Material 1

## Data Availability

All data used in this project are open to the public and can be downloaded from https://mics.unicef.org/surveys.

## Footnotes

1 No specific reason was provided in the report for the oversampling of the above-mentioned regions.

2 Number of children ages 0–4 in 2011 comes from the 2010 Ghana Population and Housing Census (data provided through IPUMS), while the number in 2017/18 comes from a 2020 population projection from the Ghana Statistical Service. These are the data sources closest in year to the survey years that are publicly available and provide age breakdown of absolute population sizes. Using the figures from 2010, there were 3,400,452 children aged 0–4 in Ghana, and 541,352 have been home alone in the previous 7 days (15.92% according to MICS 4). Using the figures from 2020, there were 4,277,603 children aged 0–4, and 1,043,735 have been home alone in the previous 7 days (24.41% according to MICS 6). Subtracting 541,352 from 1,043,735 yields 502,383 (absolute increase in number of children who have been home alone between MICS 4 and MICS 6.
