# Supplementary material for: The Increasing Prevalence of Children Home Alone in Ghana: The Importance of Considering Regional Inequalities
Source: Child Indic Res. 2023 May 27;16(5):2013–32. doi: 10.1007/s12187-023-10038-w (PMC10497642; doi:10.1007/s12187-023-10038-w)
Supplement: Supplementary file 1 — Supplementary Material 1 [file 12187_2023_10038_MOESM1_ESM.docx]

**Supplementary Material**

**Table S1.** Weighted mixed-effects Poisson regression results estimating incidence rate ratios (IRR) of number of days children were left home alone in the past 7 days, stratified by age group and survey rounds, Ghana MICS 4 (2011) and MICS 6 (2017/2018)

|  | **Model 1** | **Model 2** | **Model 3** | **Model 4** |
| --- | --- | --- | --- | --- |
|  | Ages 0-2 | Ages 0-2 | Ages 3-4 | Ages 3-4 |
|  | MICS 4 | MICS 6 | MICS 4 | MICS 6 |
| Male child (ref. female) | 0.83 | 1.18 | 1.22* | 1.06 |
|  | (0.64, 1.07) | (0.96, 1.44) | (1.00, 1.49) | (0.93, 1.21) |
| Age of child (months) | 1.06** | 1.04** | 1.02 | 1.01 |
|  | (1.02, 1.09) | (1.01, 1.06) | (1.00, 1.04) | (0.99, 1.02) |
| Child breastfeeds | 0.93 | 0.84 |  |  |
|  | (0.59, 1.47) | (0.59, 1.20) |  |  |
| Child attends education program |  |  | 1.12 | 0.92 |
|  |  |  | (0.82, 1.52) | (0.78, 1.09) |
| Urban residence (ref. rural) | 1.20 | 0.87 | 0.87 | 0.79 |
|  | (0.68, 2.10) | (0.60, 1.27) | (0.49, 1.54) | (0.50, 1.23) |
| Wealth index quintile of household (ref. Quintile 1, poorest) | | |  |  |
| Quintile 2 | 0.79 | 0.84 | 0.88 | 0.90 |
|  | (0.55, 1.14) | (0.66, 1.05) | (0.68, 1.15) | (0.74, 1.09) |
| Quintile 3 | 0.91 | 0.94 | 1.08 | 0.91 |
|  | (0.58, 1.42) | (0.54, 1.62) | (0.73, 1.60) | (0.72, 1.14) |
| Quintile 4 | 0.90 | 0.92 | 0.83 | 0.86 |
|  | (0.44, 1.83) | (0.70, 1.21) | (0.47, 1.46) | (0.68, 1.10) |
| Quintile 5, richest | 0.83 | 0.95 | 0.80 | 0.74 |
|  | (0.45, 1.55) | (0.56, 1.61) | (0.50, 1.27) | (0.50, 1.09) |
| Mother's education (years) | 0.99 | 1.00 | 0.98 | 1.00 |
|  | (0.95, 1.03) | (0.98, 1.02) | (0.95, 1.01) | (0.97, 1.03) |
| Father's education (years) | 1.00 | 0.96*** | 0.99 | 0.98 |
|  | (0.96, 1.03) | (0.94, 0.98) | (0.94, 1.03) | (0.96, 1.01) |
| Number of household members ages 5-17 | 1.03 | 1.03 | 0.95 | 1.04 |
|  | (0.98, 1.08) | (0.98, 1.08) | (0.91, 1.00) | (0.97, 1.11) |
| Number of household members ages 18+ | 1.03 | 1.03 | 1.07 | 1.00 |
|  | (0.94, 1.13) | (0.98, 1.07) | (0.97, 1.17) | (0.95, 1.05) |
| 2017/18 (ref. 2011) |  |  |  |  |
|  |  |  |  |  |
| *Strata-level variance (standard deviation)* | 0.30 (0.23) | 0.08 (0.03) | 0.40 (0.16) | 0.28 (0.12) |
| **N** | **4,460** | **5,156** | **3,062** | **3,629** |

**Notes** Results presented in incidence rate ratio (IRR) form, 95% confidence interval in parentheses; * p < 0.05, ** p < 0.01, *** p < 0.001; all models include strata-level mixed effects

**Table S2. Weighted mixed-effects Poisson regression results estimating incidence rate ratios (IRR) of number of days children were left home alone in the past 7 days, with or without regional-level poverty rate, Ghana MICS 4 (2011) and MICS 6 (2017/2018)**

|  | **Model 1** | **Model 2** | **Model 3** | **Model 4** |
| --- | --- | --- | --- | --- |
|  | Ages 0-2 | Ages 0-2 | Ages 3-4 | Ages 3-4 |
| Male child (ref. female) | 1.02 | 1.03 | 1.12 | 1.12 |
|  | (0.85, 1.21) | (0.86, 1.23) | (0.98, 1.30) | (0.97, 1.29) |
| Age of child (months) | 1.04*** | 1.04*** | 1.01 | 1.01 |
|  | (1.03, 1.06) | (1.03, 1.06) | (1.00, 1.02) | (1.00, 1.02) |
| Child breastfeeds | 0.88 | 0.88 |  |  |
|  | (0.69, 1.12) | (0.69, 1.13) |  |  |
| Child attends education program |  |  | 1.17 | 1.13 |
|  |  |  | (0.90, 1.53) | (0.86, 1.48) |
| Urban residence (ref. rural) | 0.99 | 23.76** | 0.78 | 0.28 |
|  | (0.74, 1.32) | (2.25, 251.4) | (0.58, 1.05) | (0.02, 4.48) |
| Wealth index quintile of household (ref. Quintile 1, poorest) | | |  |  |
| Quintile 2 | 0.87 | 0.82 | 0.91 | 0.90 |
|  | (0.70, 1.08) | (0.67, 1.01) | (0.78, 1.07) | (0.77, 1.05) |
| Quintile 3 | 0.97 | 0.92 | 1.01 | 0.98 |
|  | (0.72, 1.32) | (0.67, 1.27) | (0.79, 1.28) | (0.79, 1.22) |
| Quintile 4 | 0.98 | 0.92 | 0.86 | 0.85 |
|  | (0.69, 1.38) | (0.66, 1.30) | (0.66, 1.12) | (0.65, 1.11) |
| Quintile 5, richest | 0.98 | 0.92 | 0.75 | 0.75 |
|  | (0.71, 1.35) | (0.66, 1.28) | (0.54, 1.05) | (0.54, 1.03) |
| Mother's education (years) | 0.99 | 0.99 | 0.99 | 1.00 |
|  | (0.97, 1.01) | (0.97, 1.01) | (0.97, 1.02) | (0.97, 1.02) |
| Father's education (years) | 0.98 | 0.98 | 0.98 | 0.98 |
|  | (0.95, 1.00) | (0.95, 1.00) | (0.96, 1.01) | (0.96, 1.01) |
| Number of household members ages 5-17 | 1.02 | 1.03 | 1.00 | 1.01 |
|  | (0.98, 1.07) | (0.98, 1.07) | (0.95, 1.06) | (0.96, 1.06) |
| Number of household members ages 18+ | 1.03 | 1.02 | 1.02 | 1.02 |
|  | (0.99, 1.07) | (0.99, 1.06) | (0.97, 1.07) | (0.97, 1.07) |
| 2017/18 (ref. 2011) | 1.53* | 1.20 | 1.64** | 1.26 |
|  | (1.10, 2.13) | (0.95, 1.53) | (1.20, 2.25) | (0.93, 1.72) |
| 2017/18 * attends education program |  |  | 0.74 | 0.81 |
|  |  |  | (0.54, 1.01) | (0.59, 1.10) |
| *Strata-level variance (standard deviation)* | 0.07** (0.02) | 5.62* (2.25) | 0.26* (0.12) | 5.67 (4.74) |
| *Variance in regional-level poverty rate (standard deviation)* | | 0.009 (0.006) |  | 0.006 (0.004) |
| **N** | **9616** | **9616** | **6691** | **6691** |

**Notes** Results presented in incidence rate ratio (IRR) form, 95% confidence interval in parentheses; * p < 0.05, ** p < 0.01, *** p < 0.001; all models include strata-level mixed effects

Data on regional level poverty rate come from Cooke et al. (2016) for 2013 (mapped onto observations from 2011) and The World Bank (2018)
